# When Mitral Repair Fails: Understanding Recurrence, Risk Factors, and Treatment Choices

**DOI:** 10.3390/jcdd13050189

**Published:** 2026-04-29

**Authors:** Elisa Mikus, Mariafrancesca Fiorentino, Diego Sangiorgi, Niki Bernardoni, Roberto Nerla, Simone Calvi, Elena Tenti, Fausto Castriota, Carlo Savini

**Affiliations:** 1 Cardiovascular Department, Maria Cecilia Hospital, GVM Care & Research, 48033 Cotignola, Italy; 2 Department of Experimental Diagnostic and Surgical Medicine (DIMEC), University of Bologna, 40138 Bologna, Italy

**Keywords:** mitral valve repair or replacement, reoperation, minimally invasive cardiac surgery, time to failure

## Abstract

**Background:** Reintervention after mitral valve repair represents a relevant clinical challenge, yet the mechanisms and timing of repair failure remain incompletely defined. Understanding how the interval between index repair and reoperation affects failure mechanisms and the feasibility of repeat repair may help refine surgical strategies. **Methods:** We retrospectively analyzed 194 patients undergoing repeat mitral valve surgery between 2010 and 2025 after prior repair. Median age was 70 years and 61.3% were male. Patients were stratified by time to reoperation: 0–5 years (n = 91), 6–10 years (n = 42), and >10 years (n = 61). Median left ventricular ejection fraction was 58%, atrial fibrillation prevalence 32.5%, minimally invasive approach 21.6%, and EuroSCORE II 4.8%. **Results:** Baseline characteristics and operative risk were comparable across groups. However, mechanisms of repair failure differed significantly. Early failures were more commonly due to recurrent leaflet prolapse (47.8%), whereas late failures showed a higher incidence of mitral stenosis (63.9%). The rate of repeat mitral repair decreased over time, being higher in early failures compared with intermediate and late failures (17.6% vs. 14.3% vs. 8.2%). **Conclusions:** Timing of mitral repair failure is associated with distinct mechanisms and influences surgical management. Early failures are more frequently related to prolapse recurrence and are more amenable to re-repair, whereas late failures are characterized by structural degeneration and more often require valve replacement.

## 1. Introduction

Mitral valve (MV) repair is widely recognized as the treatment of choice for patients with degenerative mitral regurgitation [1], as it preserves the native valve apparatus and is associated with improved survival, better preservation of left ventricular function, and lower rates of valve-related complications compared with valve replacement [2,3,4]. Over the past decades, continuous refinements in surgical techniques, a deeper understanding of mitral valve anatomy and pathophysiology, and the development of adjunctive technologies have significantly improved the reproducibility and durability of mitral valve repair. Consequently, repair rates have progressively increased in specialized centers, with excellent early and long-term outcomes consistently reported in contemporary surgical series [5,6]. Large registry analyses and institutional experiences have confirmed that mitral valve repair can be achieved in the vast majority of patients with degenerative mitral valve disease when treated in high-volume reference centers. In particular, specialized programs have demonstrated that repair rates approaching 100% are feasible in patients with mitral valve prolapse, further supporting the role of repair as the preferred surgical strategy in this population [5,7,8].

Despite these favorable outcomes, mitral valve repair is not exempt from failure. Even in experienced institutions, recurrent mitral valve dysfunction requiring reintervention may occur over time. Previous studies have reported an inherent linearized failure rate ranging from approximately 0.5% to 4% per year following mitral valve repair [3,4,6]. Similarly, the risk of reoperation after repair has been estimated at approximately 0.5–1.5% per year in long-term follow-up analyses [9].

While several studies have described the outcomes of reoperative mitral valve surgery, less is known about how the interval between the index repair and reintervention is associated with different patterns of failure and may reflect the underlying mechanism of mitral valve dysfunction, thereby potentially influencing the subsequent surgical strategy. In particular, it remains unclear whether patients presenting with early repair failure represent a distinct clinical entity compared with those presenting later, or whether similar pathological mechanisms and risk profiles are shared across different time intervals. Understanding the causes and mechanisms underlying repair failure is essential for improving the durability of the initial procedure and for guiding the optimal management of patients requiring reintervention. The mechanisms responsible for recurrent mitral valve dysfunction after repair are heterogeneous and may include recurrent leaflet prolapse, annular dehiscence, leaflet restriction or retraction, and, less frequently, infective endocarditis.

In this context, the present study aims to describe the clinical characteristics, mechanisms of failure, and surgical management of patients undergoing reoperation after previous mitral valve repair, with particular focus on the relationship between the timing of repair failure and the underlying mechanism of mitral valve dysfunction. Additionally, we sought to evaluate the feasibility of repeat mitral valve repair and the role of minimally invasive surgical approaches in this complex clinical setting.

## 2. Materials and Methods

### 2.1. Study Population

All adult patients (≥18 years) who underwent repeat mitral valve surgery at our institution between January 2010 and December 2025 following a previous mitral valve repair were retrospectively included in the present analysis. No formal sample size calculation was performed; instead, all consecutive eligible cases during the study period were considered. Patients were included if they had previously undergone surgical mitral valve repair and subsequently required a second mitral valve intervention for recurrent valve dysfunction or other mitral valve-related pathology. To minimize potential sources of bias and to focus specifically on non-infective mechanisms of repair failure, all patients undergoing reoperation for mitral valve dysfunction related to active or previous infective endocarditis were excluded from the analysis. During the study period, a total of 4698 mitral valve procedures were performed at our center. Among these, 194 patients required reoperation after a prior mitral valve repair and constituted the study population. Importantly, this cohort also included patients whose index mitral valve repair had been performed at other institutions and, in some cases, patients with a history of additional previous cardiac surgical procedures. Consequently, the total number of patients initially undergoing mitral valve repair from which these reoperations derived cannot be precisely defined.

**Figure 1:**
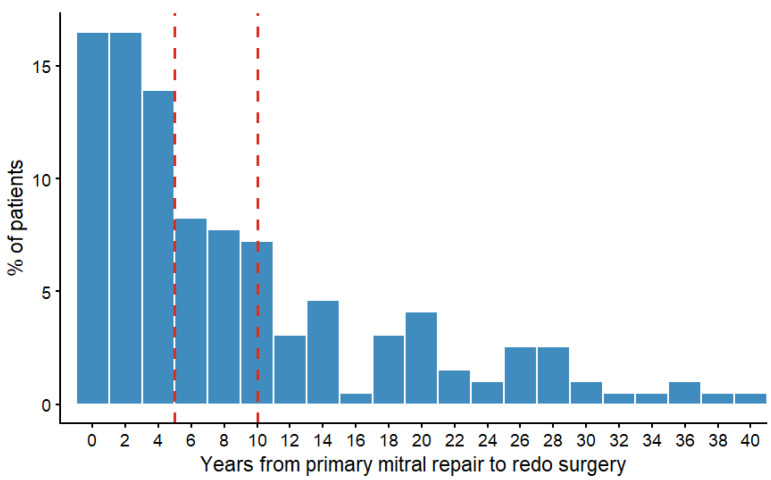
Distribution of time to reoperation. The vertical red lines identify the chosen cut-offs for early, mid and late failure.

Patients were stratified into three groups according to the interval between the index repair and the reintervention: early failure (0–5 years, n = 91), intermediate failure (6–10 years, n = 42), and late failure (>10 years, n = 61). Median time to reoperation was 6.3 years, ranging from 30 day to 53 years (25th–75th percentiles of 2.2–13.7 years) (Figure 1). The choice of surgical approach, including conventional sternotomy or minimally invasive surgery, as well as the decision to perform repeat mitral valve repair (re-repair) or valve replacement, was made at the discretion of the operating surgeon based on preoperative imaging findings, intraoperative assessment of valve anatomy, and overall patient characteristics.

The study was conducted in accordance with the principles of the Declaration of Helsinki. Ethical approval was obtained from the Romagna Ethics Committee (Prot. 9689/2019 I.5/186). Given the retrospective design of the study, the requirement for individual informed consent was waived.

Preoperative, intraoperative, and postoperative clinical data were collected from institutional medical records. Variables analyzed included demographic characteristics, comorbidities, operative risk assessed by EuroSCORE II, mechanism of mitral repair failure, surgical strategy (re-repair vs. valve replacement), and operative approach (minimally invasive vs. sternotomy). Postoperative outcomes included in-hospital mortality—defined as any death occurring before discharge from the index hospitalization—and the incidence of major postoperative complications.

The primary endpoint of the study was to evaluate the relationship between the timing of mitral valve repair failure and the underlying mechanism of valve dysfunction leading to reintervention. Secondary endpoints included the surgical management strategy adopted at reoperation—specifically the rate of repeat mitral valve repair versus valve replacement—as well as the use of minimally invasive approaches and the incidence of in-hospital mortality and major postoperative complications.

### 2.2. Statistical Analysis

After check normal distribution with Shapiro–Wilk test, continuous variables were presented as medians with interquartile ranges (IQR), categorical variables were expressed as absolute numbers and frequencies. Univariable logistic regression was conducted to assess the relation between in-hospital outcomes and timing of mitral valve repair failure. Given the low number of events, the events-per-variable ratio was insufficient to support a reliable multivariable model. All analyses were performed using R version 4.5.0 (R Foundation for Statistical Computing, Vienna, Austria), with *p*-values < 0.05 considered as statistically significant. ChatGPT 5.0 was used for code debugging.

## 3. Results

### 3.1. Patients’ Characteristics

Baseline demographic and clinical characteristics of the study population are reported in Table 1 and compared across the three groups defined according to the interval between the index mitral valve repair and the reintervention. The overall cohort had a median age of 70 years (IQR 59–75), and the majority of patients were male (61.3%).

No statistically significant differences were observed among the three groups with regard to the main preoperative clinical variables. In particular, age (*p* = 0.357), sex distribution (*p* = 0.348), body mass index (BMI) (*p* = 0.424), New York Heart Association (NYHA) functional class (*p* = 0.107), and left ventricular ejection fraction (*p* = 0.767) were comparable across the early (0–5 years), intermediate (6–10 years), and late (>10 years) failure groups. Similarly, operative risk as assessed by EuroSCORE II did not significantly differ between groups (*p* = 0.054), indicating a relatively homogeneous preoperative risk profile among patients undergoing reintervention at different time intervals.

The only significant difference observed among the three groups concerned the prevalence of preoperative atrial fibrillation, which was significantly higher in patients presenting with late mitral repair failure (>10 years) (*p* = 0.002).

**Figure 2:**
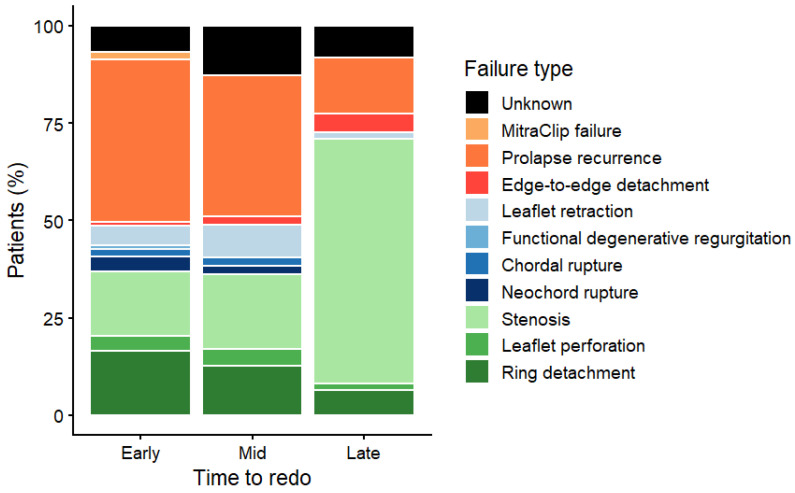
Mechanisms of mitral valve repair failure according to time to reoperation.

Relevant differences between groups emerged when analyzing echocardiographic findings and the mechanisms of mitral valve repair failure, as summarized in Table 2 and Figure 2.

Patients presenting with early failure (<5 years) showed a higher incidence of recurrent leaflet prolapse (*p* < 0.001). Conversely, patients presenting with late failure (>10 years) more commonly exhibited progressive evolution toward mitral valve stenosis, reflecting long-term structural changes in the valve apparatus (*p* < 0.001).

### 3.2. Intraoperative Characteristics

Intraoperative data are summarized in Table 3. The overall rate of repeat mitral valve repair (re-repair) was low, at 13.9%, without reaching statistical significance among the three groups (*p* = 0.246). However, a trend was observed suggesting that re-repair was more likely in patients with early failure compared with those presenting with late failure. Minimally invasive surgery via right minithoracotomy was performed in 21.6% of patients, with a similar distribution across the three groups (*p* = 0.139). Among patients undergoing mitral valve replacement, biological prostheses were more commonly used than mechanical valves (n = 94 vs. n = 73), although no statistically significant differences were observed between groups for either type (biological *p* = 0.696, mechanical *p* = 0.885).

Median aortic cross-clamp and cardiopulmonary bypass (CPB) times for the overall cohort were 83 min (IQR 66–109, *p* = 0.427) and 112 min (IQR 85–138, *p* = 0.792), respectively, with no significant differences among the three groups. Concomitant procedures were relatively uncommon, and mainly included closure of the left atrial appendage (8.8%, predominantly in the late failure group, *p* < 0.001), tricuspid valve repair (17.0%, mostly in the late failure group, *p* = 0.031), aortic valve replacement (10.8%) and CABG (3.1%).

### 3.3. In-Hospital Outcomes

In-hospital outcomes for the overall cohort and their stratification by timing of mitral repair failure are reported in Table 4. The overall in-hospital mortality was 2.1%, with no significant differences among the early (0–5 years), intermediate (6–10 years), and late (>10 years) groups (*p* = 0.556). Major postoperative complications—including prolonged mechanical ventilation (*p* = 0.509), reintubation (*p* = 0.170) and stroke (*p* = 1.000) were not affected by time to redo, with no statistically significant differences observed.

The only difference, in terms of time to redo, was new-onset postoperative atrial fibrillation (*p* = 0.042). Other adverse events, including sepsis (*p* = 0.256), need for pacemaker implantation (*p* = 0.390), and re-thoracotomy for bleeding (*p* = 0.592), occurred at low rates and were evenly distributed among the three groups. Median ventilation time for the overall cohort was 8 h, median ICU stay was 2 days, and median postoperative length of stay was 8 days, without significant variation among groups.

### 3.4. Repair or Replacement

**Figure 3:**
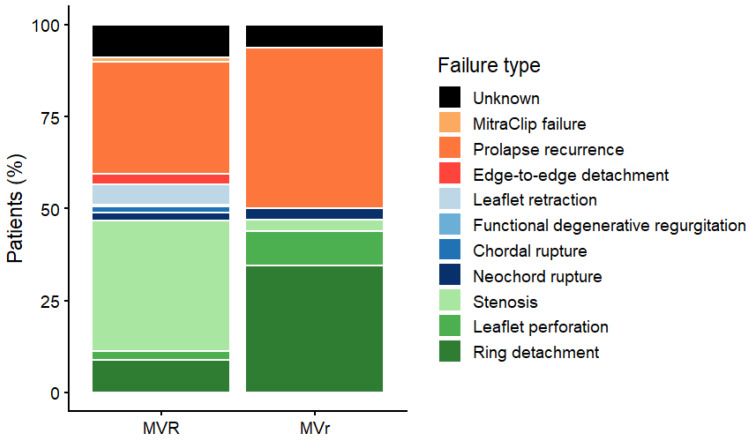
Mechanisms of mitral valve repair failure according to repair/replace.

We further analyzed the cohort by comparing patients who underwent repeat mitral valve repair (re-repair) with those who required mitral valve replacement at reoperation. Re-repair was performed more frequently in younger patients (median 66 (52–71) vs. 70 (60–76), *p* = 0.014) and tended to occur in those presenting with earlier failure of the initial mitral repair, with a shorter median interval between the index procedure and reoperation (4.27 years (0.63–8.29) vs. 6.58 years (2.29–14.24), *p* = 0.060). In this subgroup, the predominant mechanism of failure was recurrent leaflet prolapse, most commonly related to prior implantation of artificial neochordae. Consistently, re-repair was significantly more frequent when the underlying cause of failure was recurrence of mitral prolapse, confirming that this mechanism represents the most favorable anatomical substrate for valve preservation. Conversely, patients presenting with late failure (>10 years) or with structural alterations such as leaflet retraction or progressive mitral stenosis were more likely to undergo valve replacement (Figure 3). From a technical standpoint, patients undergoing re-repair were more frequently treated through a minimally invasive right minithoracotomy approach compared with those undergoing valve replacement (44.4% vs. 18.0%, *p* = 0.004). In terms of postoperative outcomes, patients treated with re-repair required significantly fewer blood transfusions (26.9% vs. 68.3%, *p* < 0.001) and had shorter mechanical ventilation hours (5 (4, 6) vs. 9 (5, 17), *p* < 0.001). No in-hospital deaths occurred in the re-repair group, whereas the mortality rate among patients undergoing mitral valve replacement was 2.4%.

## 4. Discussion

The present study aimed to characterize patients undergoing reoperation after previous mitral valve repair and to investigate whether the timing of repair failure influences the mechanisms of recurrent mitral valve dysfunction, the surgical strategy adopted at reintervention, and the feasibility of repeat mitral valve repair. In particular, we sought to better understand whether early and late failures represent distinct clinical entities and how these differences may affect surgical decision-making.

### 4.1. When and Whether to Perform Mitral Valve Re-Repair

Historically, reoperation after failed mitral valve repair has often been managed with valve replacement rather than repeat repair. Early surgical experiences raised concerns regarding the durability of a second repair and the quality of residual valve tissue, leading many surgeons to favor prosthetic valve replacement as the most definitive treatment strategy. In the largest early series, the rate of repeat repair ranged between 16% and 49%, reflecting a relatively conservative attitude toward valve preservation in the setting of repair failure [9,10,11,12,13,14,15,16]. These concerns led many surgeons to consider valve replacement the safest and most definitive solution when facing recurrent mitral dysfunction after a previous repair [9].

Over time, however, improvements in surgical techniques and a deeper understanding of mitral valve pathology have significantly changed this paradigm. Several contemporary studies have demonstrated that repeat mitral valve repair can be performed safely and effectively in selected patients, often providing excellent outcomes while preserving the native valve apparatus [10,11,12,13]. In particular, Anyanwu and colleagues reported that re-repair can be successfully achieved in the majority of patients undergoing reoperation after failed repair when the underlying mechanism remains amenable to reconstruction [10]. Similarly, Aphram and colleagues highlighted the importance of identifying the specific cause of repair failure in order to tailor the surgical strategy and maximize the feasibility of valve preservation [11].

More recent data further reinforce this repair-oriented strategy. In a contemporary study including 330 patients undergoing reoperation after degenerative mitral valve repair, Moore and colleagues demonstrated that repeat mitral valve repair can be performed with very low operative mortality and favorable perioperative outcomes. Importantly, the authors emphasized that the mechanisms of early repair failure are generally more suitable for re-repair compared with those observed in late failures [17]. Similar conclusions have been reported in other recent series evaluating redo mitral valve surgery, confirming that repeat repair can be a safe and effective strategy when the anatomical substrate remains favorable [18,19].

The findings of the present study are consistent with this growing body of evidence. In our cohort, repeat mitral valve repair was more frequently performed in younger patients and tended to occur in those presenting with earlier failure of the index repair. Moreover, the predominant mechanism of failure in patients undergoing re-repair was recurrent leaflet prolapse, most commonly associated with prior implantation of artificial neochordae. This mechanism represents one of the most favorable anatomical substrates for repeat repair, as the leaflet tissue is generally preserved and amenable to further reconstructive techniques. Conversely, patients presenting with late repair failure were more likely to exhibit structural changes affecting the mitral valve apparatus, including leaflet fibrosis, retraction, or progressive mitral stenosis. In these situations, valve replacement was more frequently required because structural remodeling of the leaflets may limit the feasibility of a durable reconstructive procedure. These findings are consistent with previous studies describing mitral stenosis after repair as a particularly challenging condition, frequently requiring valve replacement due to irreversible leaflet alterations [20]. Taken together, these observations reinforce the concept that the feasibility of repeat repair is primarily determined by the underlying mechanism of valve dysfunction rather than by the simple occurrence of reoperation. Careful preoperative assessment and intraoperative evaluation of valve anatomy remain essential in determining the most appropriate surgical strategy.

### 4.2. Early Versus Late Mitral Valve Repair Failure

Another important finding of the present study concerns the relationship between the timing of repair failure and the underlying pathological mechanisms of mitral valve dysfunction. Our results demonstrate that early and late failures represent distinct clinical entities characterized by different mechanisms of valve failure. Importantly, the interval between the index repair and reoperation should not be interpreted as a causal factor influencing the mechanism of failure; rather, it is more appropriately viewed as a marker reflecting different underlying pathophysiological processes.

Early failures were predominantly associated with recurrent leaflet prolapse. This observation suggests that early recurrence of mitral regurgitation may more often be related to technical or procedural factors from the index operation, such as suboptimal neochordal length adjustment or incomplete correction of the initial leaflet pathology or ring detachment. In contrast, late failures were more commonly associated with progressive structural alterations of the mitral valve apparatus, including leaflet fibrosis, retraction, and evolution toward mitral stenosis. These findings likely reflect long-term remodeling processes affecting the repaired valve as well as the natural progression of degenerative valve disease. The high prevalence of mitral stenosis observed in this subgroup suggests that this condition is not merely the result of generic degenerative changes, but rather the consequence of a complex interplay between anatomical remodeling and procedure-related factors. From a pathophysiological standpoint, several mechanisms may contribute to the development of post-repair mitral stenosis over time. Annuloplasty-related factors likely play a central role: the use of undersized or rigid complete rings may lead to a progressive reduction in the effective mitral valve area, particularly when combined with long-term changes in annular dynamics. In addition, leaflet and subvalvular remodeling—characterized by fibrosis, thickening, and reduced mobility—may progressively impair diastolic opening of the valve. Progressive calcification of both the mitral annulus and the leaflets may further exacerbate this process, leading to increased stiffness and restricted motion. Moreover, the evolution of surgical techniques over the past decades should also be considered. Earlier repair strategies often relied on more aggressive leaflet resection and less physiologic annuloplasty concepts, which may predispose to reduced leaflet excursion and a higher risk of functional stenosis in the long term compared with contemporary repair approaches that aim to preserve leaflet tissue and maintain annular dynamics. Taken together, these mechanisms likely act synergistically over time, ultimately leading to the progressive development of a stenotic mitral valve physiology in patients presenting with late repair failure. A similar distinction between early and late repair failure has been described in the recent literature. Moore and colleagues demonstrated that early failures are typically characterized by residual or recurrent leaflet prolapse, whereas late failures are more frequently associated with progressive degenerative changes and structural remodeling of the valve apparatus [17]. These differences are clinically relevant because they directly influence the likelihood of successful repeat repair. The greater repairability observed in early failures may also be explained by the absence of significant fibrotic remodeling or leaflet scarring, which are more commonly observed in late failures and may significantly limit reconstructive options. Consequently, early recognition of recurrent mitral regurgitation and timely referral for reoperation may increase the likelihood of successful valve preservation. Finally, it should be emphasized that the lack of detailed and standardized information regarding the index mitral valve repair—particularly the specific surgical techniques employed—represents a relevant limitation, preventing a direct correlation between initial repair strategy and mechanism of failure. These findings should therefore be interpreted as observed associations rather than evidence of a causal relationship between timing of failure and underlying mechanism, as they may be significantly influenced by unmeasured factors related to the index repair, including surgical technique and procedural quality.

### 4.3. Role of Minimally Invasive Surgery

Minimally invasive mitral valve surgery has progressively gained acceptance as an alternative to conventional median sternotomy, offering potential advantages such as reduced surgical trauma, faster recovery, and improved postoperative comfort. Although redo mitral valve surgery has traditionally been considered technically demanding, advances in surgical techniques and increasing experience have expanded the applicability of minimally invasive approaches even in reoperative settings [21,22]. In the present study, minimally invasive surgery via right minithoracotomy was used in approximately one fifth of patients undergoing redo mitral valve surgery. Interestingly, patients undergoing repeat mitral valve repair were significantly more likely to be treated through a minimally invasive approach compared with those undergoing valve replacement. This observation likely reflects the more favorable anatomical conditions encountered in patients with recurrent prolapse, where valve tissue quality remains suitable for reconstructive techniques and operative exposure can be safely achieved through a minimally invasive access. Conversely, more complex pathological scenarios, such as severe leaflet fibrosis or mitral stenosis, may require more extensive surgical exposure and are therefore more frequently managed through conventional sternotomy. The higher proportion of minimally invasive procedures observed in patients undergoing re-repair likely reflects, at least in part, a selection process. In our cohort, patients eligible for repeat repair more frequently presented with early failure and favorable anatomical substrates, most commonly recurrent leaflet prolapse with preserved leaflet tissue and limited structural remodeling, which are inherently more suitable for both valve preservation and minimally invasive access. Conversely, patients requiring valve replacement more often exhibited complex pathological features that necessitated wider surgical exposure and therefore favored a conventional sternotomy. Nevertheless, it is also conceivable that the minimally invasive approach itself may facilitate re-repair in selected cases. The enhanced visualization of the mitral valve through a targeted left atrial access, together with the reduced surgical trauma, may support reconstructive strategies, particularly in reoperative settings. Therefore, the association between minimally invasive surgery and re-repair observed in this study likely reflects a combination of patient selection and procedural advantages, rather than a direct causal relationship. In addition, the choice of surgical access at the index operation may have important implications for subsequent reoperations. When the primary mitral procedure is performed through a minimally invasive right minithoracotomy, a potential advantage at reoperation is the possibility of performing a full sternotomy in a “virgin” mediastinum, thereby reducing the technical challenges and risks associated with redo sternotomy. Conversely, in patients who initially underwent surgery via median sternotomy, a minimally invasive approach at reoperation may be advantageous by avoiding re-entry through dense mediastinal adhesions and limiting surgical dissection to a more targeted access [21]. This complementary relationship between surgical approaches suggests that minimally invasive surgery may offer strategic advantages not only in the primary setting but also across the entire surgical lifetime of the patient, potentially facilitating safer and more flexible planning of redo procedures.

### 4.4. Clinical Implications

The findings of this study provide several clinically relevant insights. First, they confirm that redo surgery after mitral valve repair can be performed with acceptable perioperative outcomes. Second, they demonstrate that the timing of repair failure is closely associated with distinct mechanisms of valve dysfunction that strongly influence the feasibility of repeat repair. In particular, recurrent prolapse represents the mechanism most amenable to valve preservation and should prompt careful consideration of repeat repair, particularly in younger patients and in those presenting with early failure of the index procedure. Conversely, patients presenting with late failure associated with structural leaflet remodeling or mitral stenosis may more frequently require valve replacement. These observations support a mechanism-based approach to the management of failed mitral valve repair and highlight the importance of comprehensive preoperative evaluation and individualized surgical planning.

### 4.5. Limitations

This study has several limitations that should be acknowledged. First, its retrospective and observational design inherently carries the risk of selection and information bias. Furthermore, the analysis reflects the experience of a single institution, which may limit the generalizability of the findings to other centers with different patient populations, surgical expertise, and institutional practices. Another important limitation is related to the nature of the study population. A substantial proportion of patients included in the analysis had undergone their initial mitral valve repair at other institutions and, in some cases, had a history of additional prior cardiac surgical procedures. Consequently, the original population from which these reoperations derived cannot be precisely defined. In addition, detailed and standardized information regarding the index mitral valve repair—such as the specific surgical techniques employed, intraoperative findings, and immediate postoperative echocardiographic results—was not consistently available. This limitation prevented a reliable analysis of the relationship between the initial repair strategy and the subsequent mechanism of failure, and did not allow us to confirm whether specific techniques (e.g., neochordae implantation, leaflet resection, or annuloplasty strategy) were associated with different patterns of failure. Therefore, any pathophysiological interpretation linking timing of failure to underlying mechanisms should be considered hypothesis-generating rather than definitive. This limitation may have influenced the ability to fully characterize the mechanisms of repair failure. In addition, the decision to perform repeat mitral valve repair versus valve replacement, as well as the choice of surgical approach (conventional sternotomy versus minimally invasive surgery), was left to the discretion of the operating surgeon and was based on preoperative imaging, intraoperative findings, and individual surgical expertise. This non-standardized decision-making process may have introduced treatment selection bias. The present study also focused primarily on early postoperative outcomes and mechanisms of repair failure. Long-term clinical and echocardiographic follow-up after reintervention—including valve durability, recurrence of mitral dysfunction, and late survival—was not systematically assessed and therefore could not be analyzed. Finally, although patients were stratified according to the time interval between the index repair and reoperation, the relatively limited sample size within each subgroup may have reduced the statistical power to detect smaller differences between groups; in particular, the relatively small number of outcome events did not allow for multivariable adjustment, limiting the ability to control for potential confounding factors. As a result, the associations observed in univariable analyses should be interpreted with caution and cannot be considered causal.

Future prospective, multicenter studies with standardized data collection and extended follow-up will be necessary to better define the mechanisms, timing, and optimal management strategies for mitral valve repair failure.

## 5. Conclusions

Redo surgery after mitral valve repair can be performed with low in-hospital mortality and acceptable perioperative outcomes. The timing of repair failure is strongly associated with distinct mechanisms of mitral valve dysfunction. Early failures are predominantly characterized by recurrent leaflet prolapse and are more frequently amenable to repeat mitral valve repair, whereas late failures are more often associated with structural leaflet remodeling or mitral stenosis and may require valve replacement. Repeat mitral valve repair was more commonly performed in younger patients and in those presenting with early recurrence of mitral regurgitation. In addition, minimally invasive surgery can be safely applied in selected patients undergoing redo mitral valve procedures. Overall, these findings support a surgical strategy that prioritizes repeat mitral valve repair whenever anatomically feasible, particularly in patients with early repair failure due to recurrent prolapse.

## Figures and Tables

**Table 1:** Preoperative characteristics.

	Overall	Early	Mid	Late	*p*
n	194	91	42	61	
Age, median (IQR)	70 (59, 75)	67 (56, 76)	71 (64, 75)	70 (61, 75)	0.357
Male, n (%)	119 (61.3)	60 (65.9)	26 (61.9)	33 (54.1)	0.348
BMI, median (IQR)	24.9 (22.8, 27.8)	24.9 (22.8, 27.8)	26.1 (23.3, 29.3)	24.6 (22.7, 27.3)	0.424
Hypertension, n (%)	114 (58.8)	52 (57.1)	24 (57.1)	38 (62.3)	0.799
Diabetes, n (%)	16 (8.2)	6 (6.6)	3 (7.1)	7 (11.5)	0.556
Dyslipidemia, n (%)	76 (39.2)	40 (44.0)	14 (33.3)	22 (36.1)	0.433
Preoperative atrial fibrillation, n (%)	63 (32.5)	20 (22.0)	13 (31.0)	30 (49.2)	0.002
Preoperative pacemaker, n (%)	10 (5.2)	5 (5.5)	2 (4.8)	3 (4.9)	1.000
NYHA class, n (%)					0.107
I	29 (15.1)	20 (22.2)	4 (9.5)	5 (8.3)	
II	90 (46.9)	37 (41.1)	25 (59.5)	28 (46.7)	
III	69 (35.9)	30 (33.3)	13 (31.0)	26 (43.3)	
IV	4 (2.1)	3 (3.3)	0 (0.0)	1 (1.7)	
LVEF, median (IQR)	58 (54, 63.75)	59.50 (54, 64)	60 (55, 62.50)	57 (51, 62)	0.767
Previous stroke, n (%)	6 (3.1)	2 (2.2)	1 (2.4)	3 (4.9)	0.576
Previous TIA, n (%)	6 (3.1)	3 (3.3)	1 (2.4)	2 (3.3)	1.000
Significant carotid disease, n (%)	1 (0.5)	1 (1.1)	0 (0.0)	0 (0.0)	1.000
Creatinine md/dL, median (IQR)	0.98 (0.82, 1.12)	1 (0.86, 1.21)	1 (0.80, 1.09)	0.96 (0.80, 1.10)	0.233
COPD, n (%)	28 (14.4)	11 (12.1)	2 (4.8)	15 (24.6)	0.013
EuroSCORE II, median (IQR)	4.80 (2.74, 8.78)	3.71 (2.28, 8.77)	4.56 (2.70, 6.91)	6.13 (3.87, 9.71)	0.054
Years between procedures, median (IQR)	6.29 (2.25, 13.70)	1.88 (0.69, 3.43)	8 (6.58, 9.04)	20.45 (14.18, 28.08)	<0.001

COPD = Chronic Obstructive Pulmonary Disease; LVEF = Left Ventricular Ejection Fraction; NYHA = New York Heart Association; TIA = Transient Ischemic Attack.

**Table 2:** Echocardiographic findings and the mechanisms of mitral valve repair failure.

	Overall	Early	Mid	Late	*p*
	194	91	42	61	
Isolated mitral valve procedure, n (%)	134 (69.4)	63 (69.2)	27 (64.3)	44 (73.3)	0.637
Combined procedure, n (%)	59 (30.6)	29 (31.9)	15 (35.7)	15 (25.0)	0.492
Annuloplasty ring diameter, n (%) (24 missing)					0.023
No	38 (22.4)	15 (17.6)	4 (10.8)	19 (39.6)	
26 mm	2 (1.2)	1 (1.2)	1 (2.7)	0 (0.0)	
28 mm	16 (9.4)	8 (9.4)	3 (8.1)	5 (10.4)	
30 mm	21 (12.4)	13 (15.3)	4 (10.8)	4 (8.3)	
32 mm	20 (11.8)	7 (8.2)	8 (21.6)	5 (10.4)	
34 mm	39 (22.9)	27 (31.8)	7 (18.9)	5 (10.4)	
36 mm	21 (12.4)	9 (10.6)	4 (10.8)	8 (16.7)	
38 mm	11 (6.5)	4 (4.7)	5 (13.5)	2 (4.2)	
40 mm	1 (0.6)	1 (1.2)	0 (0.0)	0 (0.0)	
42 mm	1 (0.6)	0 (0.0)	1 (2.7)	0 (0.0)	
Leaflet resection, n (%) (26 missing)					0.178
No	121 (72.0)	66 (76.7)	20 (58.8)	35 (72.9)	
PML	38 (22.6)	15 (17.4)	11 (32.4)	12 (25.0)	
PML + AML	1 (0.6)	0 (0.0)	1 (2.9)	0 (0.0)	
PML or AML	5 (3.0)	2 (2.3)	2 (5.9)	1 (2.1)	
AML	3 (1.8)	3 (3.5)	0 (0.0)	0 (0.0)	
Gore-Tex chordae, n (%) (26 missing)					0.145
No	108 (64.3)	51 (59.3)	21 (61.8)	36 (75.0)	
PML	30 (17.9)	20 (23.3)	5 (14.7)	5 (10.4)	
PML + AML	6 (3.6)	5 (5.8)	1 (2.9)	0 (0.0)	
PML or AML	7 (4.2)	3 (3.5)	1 (2.9)	3 (6.2)	
AML	12 (7.1)	3 (3.5)	5 (14.7)	4 (8.3)	
Edge to edge, n (%)	28 (15.6)	14 (16.1)	6 (15.8)	8 (14.8)	1.000
Reason of failure, n (%)					
Ring detachment	27 (14.0)	17 (18.9)	6 (14.3)	4 (6.6)	0.093
Prolapse recurrence	69 (35.8)	43 (47.8)	17 (40.5)	9 (14.8)	<0.001
Stenosis	65 (33.5)	17 (18.7)	9 (21.4)	39 (63.9)	<0.001
Leaflet perforation	7 (3.6)	4 (4.4)	2 (4.8)	1 (1.6)	0.684
Edge-to-edge detachment	5 (2.6)	1 (1.1)	1 (2.4)	3 (4.9)	0.302
Leaflet retraction	10 (5.2)	5 (5.5)	4 (9.5)	1 (1.6)	0.226
Unknown Previous repair failure	18 (9.3)	7 (7.7)	6 (14.3)	5 (8.2)	0.475
MitraClip failure	2 (1.0)	2 (2.2)	0 (0.0)	0 (0.0)	0.703
Functional/degenerative regurgitation	1 (0.5)	1 (1.1)	0 (0.0)	0 (0.0)	1.000
Chordal rupture	3 (1.5)	2 (2.2)	1 (2.4)	0 (0.0)	0.597
NeoChord	5 (3.0)	4 (4.7)	1 (2.9)	0 (0.0)	0.531

AML = anterior mitral leaflet; PML = posterior mitral leaflet.

**Table 3:** Intraoperative characteristics.

	Overall	Early	Mid	Late	*p*
	194	91	42	61	
Minimally invasive approach, n (%)	42 (21.6)	24 (26.4)	10 (23.8)	8 (13.1)	0.139
Cardiopulmonary bypass time, median (IQR)	112 (85, 138)	112 (81, 139)	110 (89, 137)	115 (91, 133)	0.792
Cross-clamp time, median (IQR)	83 (66, 109)	80 (63, 103)	845 (68, 117)	84 (74, 107)	0.427
Postoperative mitral valve repair (PLM), n (%)	27 (13.9)	16 (17.6)	6 (14.3)	5 (8.2)	0.246
Annuloplasty ring diameter post-repair, n (%)					0.195
No	8 (29.6)	7 (43.8)	0 (0.0)	1 (20.0)	
26 mm	1 (3.7)	0 (0.0)	1 (16.7)	0 (0.0)	
28 mm	2 (7.4)	0 (0.0)	1 (16.7)	1 (20.0)	
32 mm	3 (11.1)	2 (12.5)	1 (16.7)	0 (0.0)	
34 mm	4 (14.8)	2 (12.5)	1 (16.7)	1 (20.0)	
36 mm	6 (22.2)	4 (25.0)	1 (16.7)	1 (20.0)	
38 mm	2 (7.4)	1 (6.2)	1 (16.7)	0 (0.0)	
40 mm	1 (3.7)	0 (0.0)	0 (0.0)	1 (20.0)	
Leaflet resection post-repair, n (%)	3 (1.5)	2 (2.2)	1 (2.4)	0 (0.0)	0.597
Gore-Tex chordae post-repair, n (%)					0.734
No	179 (92.3)	80 (87.9)	40 (95.2)	59 (96.7)	
PML	9 (4.6)	5 (5.5)	2 (4.8)	2 (3.3)	
PML + AML	2 (1.0)	2 (2.2)	0 (0.0)	0 (0.0)	
AML	1 (0.5)	1 (1.1)	0 (0.0)	0 (0.0)	
NeoChord, n (%)	3 (1.5)	3 (3.3)	0 (0.0)	0 (0.0)	0.178
Bioprosthesis implantation, n (%)	94 (48.5)	45 (49.5)	22 (52.4)	27 (44.3)	0.696
Bioprosthesis diameter, n (%)					0.970
25 mm	16 (17.0)	6 (13.3)	4 (18.2)	6 (22.2)	
27 mm	32 (34.0)	17 (37.8)	7 (31.8)	8 (29.6)	
29 mm	32 (34.0)	15 (33.3)	7 (31.8)	10 (37.0)	
31 mm	13 (13.8)	6 (13.3)	4 (18.2)	3 (11.1)	
33 mm	1 (1.1)	1 (2.2)	0 (0.0)	0 (0.0)	
Mechanical valve implantation, n (%)	73 (37.6)	30 (33.0)	14 (33.3)	29 (47.5)	0.164
Mechanical valve diameter, n (%)					0.885
23 mm	3 (4.1)	1 (3.3)	1 (7.1)	1 (3.4)	
25 mm	16 (21.9)	5 (16.7)	2 (14.3)	9 (31.0)	
27 mm	18 (24.7)	7 (23.3)	5 (35.7)	6 (20.7)	
29 mm	22 (30.1)	10 (33.3)	5 (35.7)	7 (24.1)	
31 mm	12 (16.4)	6 (20.0)	1 (7.1)	5 (17.2)	
33 mm	2 (2.7)	1 (3.3)	0 (0.0)	1 (3.4)	
Left atrial appendage closure, n (%)	17 (8.8)	1 (1.1)	2 (4.8)	14 (23.0)	<0.001
Tricuspid valve repair, n (%)	33 (17.0)	11 (12.1)	5 (11.9)	17 (27.9)	0.031
Other procedures, n (%)					0.014
AVR	21 (10.8)	3 (3.3)	6 (14.3)	12 (19.7)	
CABG	6 (3.1)	5 (5.5)	0 (0.0)	1 (1.6)	
ASD/PFO closure	6 (3.1)	2 (2.2)	1 (2.4)	3 (4.9)	
Ablation	6 (3.1)	1 (1.1)	2 (4.8)	3 (4.9)	
Other	4 (2.1)	3 (3.3)	0 (0.0)	1 (1.6)	

AML = anterior mitral leaflet; PML = posterior mitral leaflet; NeoChord = neochord implantation. AVR = aortic valve replacement; CABG = Coronary Artery Bypass Grafting; ASD/PFO = defect closure (Atrial Septal Defect/Patent Foramen Ovale).

**Table 4:** In-hospital outcomes.

	Overall	Early	Mid	Late	*p*	OR (95% CI), *p*
	194	91	42	61		
Blood transfusion, n (%)	121 (62.7)	55 (61.1)	31 (73.8)	35 (57.4)	0.205	1.000 (0.974–1.027), *p* = 0.982
ICU, n (%)	0 (0.0)	0 (0.0)	0 (0.0)	0 (0.0)	1.000	/
Cardiac tamponade, n (%)	3 (1.5)	2 (2.2)	0 (0.0)	1 (1.6)	1.000	0.966 (0.839–1.112), *p* = 0.633
Severe cardiac failure, n (%)	30 (15.5)	15 (16.5)	7 (16.7)	8 (13.1)	0.871	0.980 (0.940–1.021), *p* = 0.323
Postoperative stroke, n (%)	0 (0.0)	0 (0.0)	0 (0.0)	0 (0.0)	1.000	/
Postoperative TIA, n (%)	0 (0.0)	0 (0.0)	0 (0.0)	0 (0.0)	1.000	/
Re-exploration for bleeding, n (%)	9 (4.6)	2 (2.2)	2 (4.8)	5 (8.2)	0.220	1.015 (0.961–1.072), *p* = 0.592
Acute kidney injury, n (%)	13 (6.7)	5 (5.5)	6 (14.3)	2 (3.3)	0.094	1.001 (0.951–1.053), *p* = 0.972
AKI grade 1, n (%)	9 (4.6)	3 (3.3)	5 (11.9)	1 (1.6)	0.058	0.960 (0.881–1.046), *p* = 0.354
CVVH, n (%)	6 (3.1)	3 (3.3)	1 (2.4)	2 (3.3)	1.000	1.023 (0.961–1.088), *p* = 0.476
PMK, n (%)	13 (6.7)	8 (8.8)	3 (7.1)	2 (3.3)	0.460	0.972 (0.911–1.037), *p* = 0.390
New-onset AF, n (%)	29 (14.9)	17 (18.7)	6 (14.3)	6 (9.8)	0.330	0.945 (0.894–0.998), *p* = 0.042
Respiratory failure, n (%)	14 (7.2)	8 (8.8)	1 (2.4)	5 (8.2)	0.477	1.007 (0.961–1.056), *p* = 0.764
Reintubation, n (%)	12 (6.2)	4 (4.4)	2 (4.8)	6 (9.8)	0.337	1.031 (0.987–1.076), *p* = 0.170
Gut ischemia, n (%)	0 (0.0)	0 (0.0)	0 (0.0)	0 (0.0)	1.000	/
Wound complications, n (%)	11 (5.7)	5 (5.5)	5 (11.9)	1 (1.6)	0.085	0.938 (0.854–1.030), *p* = 0.180
Sepsis, n (%)	6 (3.1)	2 (2.2)	1 (2.4)	3 (4.9)	0.576	1.034 (0.976–1.095), *p* = 0.256
Mechanical ventilation hours, median (IQR)	8 (5, 16)	7 (4, 14)	9 (5, 16)	10 (5, 18)	0.054	1.013 (0.976–1.051), *p* = 0.509
Postoperative bleeding ml, median (IQR)	450 (300, 700)	450 (300, 700)	500 (300, 750)	450 (300, 650)	0.730	/
Death at discharge, n (%)	4 (2.1)	1 (1.1)	1 (2.4)	2 (3.3)	0.556	1.054 (0.989–1.123), *p* = 0.104
ICU length of stay, median (IQR)	2 (2, 3)	2 (2, 3)	2 (2, 3)	2 (2, 3)	0.129	1.007 (0.977–1.038), *p* = 0.632
Postoperative length of stay, median (IQR)	8 (7, 11)	8 (7, 11)	9 (7, 10.75)	7 (7, 11)	0.502	/
Dialysis, n (%)	0 (0.0)	0 (0.0)	0 (0.0)	0 (0.0)	1.000	/
Preoperative MitraClip, n (%)	2 (1.0)	2 (2.2)	0 (0.0)	0 (0.0)	0.703	/

ICU = Intensive Care Unit; TIA = Transient Ischemic Attack; AKI = Acute Kidney Injury; CVVH = Continuous Veno-Venous Hemofiltration; PMK = Permanent Pacemaker; AF = atrial fibrillation.

## Data Availability

The data presented in this study are available on request from the corresponding author. The data are not publicly available due to Data Protection Directive 95/46/EC.

## Abbreviations

The following abbreviations are used in this manuscript:

AF	Atrial Fibrillation
AKI	Acute Kidney Injury
AML	Anterior Mitral Leaflet
ASD	Atrial Septal Defect
AVR	Aortic Valve Replacement
CABG	Coronary Artery Bypass Grafting
COPD	Chronic Obstructive Pulmonary Disease
CVVH	Continuous Veno-Venous Hemofiltration
ICU	Intensive Care Unit
LVEF	Left Ventricular Ejection Fraction
NYHA	New York Heart Association
PFO	Patent Foramen Ovale
PMK	Permanent Pacemaker
PML	Posterior Mitral Leaflet
TIA	Transient Ischemic Attack
